# Two in One: Recycled Cobalt Aluminate as a Pigment and Synergistic Flame-Retardant Agent for Polylactide

**DOI:** 10.1021/acsomega.4c09217

**Published:** 2025-03-10

**Authors:** Dienifer F. L. Horsth, Jamille S. Correa, Nayara Balaba, Julia de O. Primo, Fauze Jacó Anaissi, Rony Snyders, Philippe Dubois, Fouad Laoutid, Carla Bittencourt

**Affiliations:** † Chimie des Interactions Plasma-Surface (ChIPS), University of Mons, Mons 7000, Belgium; ‡ Chemistry Departament, Universidade Estadual do Centro-Oeste, Guarapuava 85040-167, Brazil; § Materia Nova Materials R&D Center, Av. Nicolas Copernic 3, Mons 7000, Belgium; ∥ Laboratory of Polymeric and Composite Materials (LPCM), University of Mons (UMONS), Mons 7000, Belgium

## Abstract

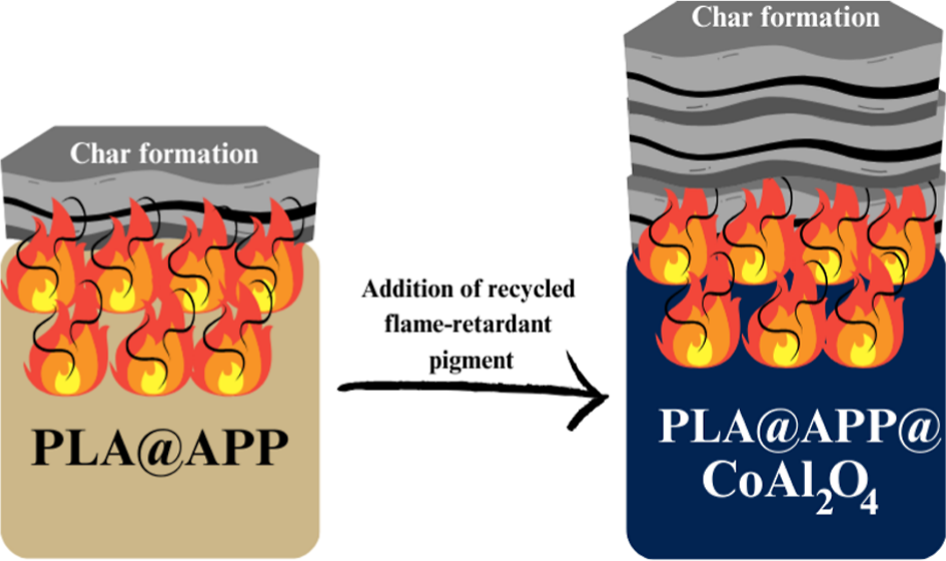

Cobalt aluminate (CoAl_2_O_4_) pigment, synthesized from recycled aluminum obtained from can seals and transformed into the boehmite phase, was combined with ammonium polyphosphate (APP422) to produce an efficient flame-retardant material for polylactide (PLA) while simultaneously imparting coloration to the polymer matrix. The chemical structure of the pigment was investigated using X-ray photoelectron spectroscopy and X-ray diffraction prior to its integration into PLA in combination with ammonium polyphosphate (APP422). Thermal gravimetric analysis highlights the superior effect of the APP422/CoAl_2_O_4_ combination that enables obtaining a greater amount of char, presenting improved thermal stability and an enhanced protective effect, as clearly evidenced by Mass Loss Cone test results. A reduction of 70% in peak heat release was observed when APP422 was combined with CoAl_2_O_4_, in contrast to a reduction of 31% when only APP422 was used at a similar incorporation level. The enhanced flame-retardant properties of the combined APP422 and CoAl_2_O_4_ additives can be attributed to a rapid formation of a homogeneous char layer at the surface of the burning material when both additives are used together. This results from the interaction between Co^3+^ and APP422, which leads to the formation of the thermally stable Co_3_(PO_4_)_2_ phase.

## 1 Introduction

The circular economy involves sustainably producing goods and services, limiting the consumption of raw materials and the production of waste. The goal is to shift from a throwaway culture to a circular economy. In this sense, aluminum cans are an excellent example of fully recyclable materials. Around 5 kilos of bauxite is saved for every kilo of secondary (recycled) aluminum. Moreover, 95% of the energy needed to produce the same quantity of primary aluminum is saved for every ton of recycled aluminum.

In a recent study concerning the color stability of blue aluminates obtained from recycling, Horsth et al.^1^ reported successful synthesis of a stable blue cobalt pigment with a spinel structure (CoAl_2_O_4_) using aluminum from recycled can seals, incorporating with 10% (w/w) of Co^2+^. This study employed 20% of chromophore ions to evaluate the reactivity of cobalt within this context. The synthesis strategy is based on doping metal oxides such as titanium, aluminum, zirconium, silicon oxide, and boehmite with chromophore ions and transition metals such as V, Cr, Mn, Fe, Co, Ni, and Cu. This approach makes it easy to vary and control the chemical composition of the pigment depending on the metal used. Composite metal oxide pigments, which incorporate multiple metal constituents, demonstrate significantly enhanced catalytic activity compared with traditional single metal oxides. These pigments exhibit superior opacity, thermal stability, infrared properties, light and weather fastness, and chemical resistance.^2,3^ Therefore, these pigments may be useful in a variety of applications, including fire protection for polymeric materials. Cobalt aluminate possesses specific properties that facilitate its function as a flame-retardant. These properties include the capacity to release metal ions, which disrupt the chemical reactions associated with combustion, thereby inhibiting fire propagation.^4^ Additionally, cobalt aluminate exhibits resistance to high temperatures, chemical stability, and low toxicity.^5^ As a coloring agent, cobalt aluminate serves as an intense blue pigment and is widely employed in the coloration of ceramic bodies, plastics, paper, paint, fibers, rubber, television tubes, glass, glazes, and porcelain enamels.^6−8^

Many polymers have low resistance to fire, prompting active research into flame retardancy to develop effective methods for reducing their fire risks. The use of flame-retardants with varied chemical structures and action mechanisms is crucial to achieving this goal. Examples include halogenated fire-retardant (FR) compounds that act in the gas phase by releasing highly reactive free radicals during their thermal decomposition that scavenge the polymer degradation radicals responsible for maintaining the combustion process.^9^ Despite their effectiveness, certain halogenated FRs have been prohibited due to environmental and health concerns.^10^ Another important group of flame-retardants includes metallic hydroxides, such as aluminum trihydroxide^11^ and magnesium dihydroxide.^12^ These additives release water during their endothermic thermal decomposition, which decelerates the thermal decomposition of the polymers. The water vapor released also helps to dilute the combustible gases generated during combustion. However, the flame-retardant effect of these hydroxides is effective only until their decomposition, as at higher temperatures, as they produce noncohesive metallic oxide powder, losing their effectiveness.

Phosphorus-containing flame-retardant compounds constitute the third important group of flame-retardants, encompassing a variety of compounds wherein phosphorus is present in different oxidation states from 0 to 5. These phosphorus-based FRs may function either in the condensed phase by forming a barrier layer or in the gas phase.

The flame-retardant properties of polymers have been extensively examined in numerous reviews and publications, highlighting the necessity for enhanced flame resistance to improve the performance of polymers in applications such as 3D printing and packaging.^13−19^ To meet increasingly rigorous fire safety regulations required for technical applications, it is often necessary to incorporate a high FR content, which can usually impact other functional characteristics of the material, such as mechanical strength, viscosity, and cost. Establishing synergistic interactions between the FR and polymers can enhance FR efficacy, reducing the need for higher incorporation rates without requiring the development of expensive novel flame-retardant compounds. Metal oxides have garnered significant attention in the field of flame retardancy, especially when combined with phosphorus-based flame-retardants that function in the condensed phase.^20−23^ This category of phosphorus-based FRs promotes the formation of a protective carbonaceous layer at the exposed surface of burning materials, shielding underlays from fire. The combination of phosphorus FRs with metal oxide promotes carbonization reactions, leading to the formation of phosphorus–metallic structures with high-temperature stability. This results in an efficient protective layer that hinders heat diffusion through the material and limits the release of combustible volatile compounds into the gas phase. The metal oxide pigments developed by Horsth et al.^1^ may prove valuable as synergistic agents with phosphorus flame-retardants in polymer matrices. These pigments contain aluminum oxide combined with another metal, potentially enabling the creation of new catalytic effects that facilitate the formation of thermally stable and homogeneous carbonaceous structures during combustion.

In this study, an ecofriendly flame-retardant was designed and synthesized through the recycling of aluminum can seals via acid digestion and the precipitation of boehmite. This process included the incorporation of cobalt ions Co^2+^ and subsequent calcination, resulting in the formation of the flame-retardant pigment. Furthermore, this study examines the potential synergistic flame-retardant effect of the cobalt aluminate pigment (CoAl_2_O_4_) derived from recycled aluminum can seals. This pigment was combined with ammonium polyphosphate (APP422) in polylactide (PLA). PLA was selected due to its growing popularity in technical applications requiring fire resistance as well as its ability to interact with ammonium polyphosphate to form a carbonaceous insulating layer. This composite was developed as a preliminary test for possible future application as a material for 3D printing, as the adoption of PLA as a preferred material in 3D printing activities continues to rise, driven by increasing demand for rapid prototyping of functional and prototype objects especially in the manufacturing sector.^24^ The primary goal of this work was to validate the dual functionality of CoAl_2_O_4_ as a pigment and flame-retardant in PLA, focusing on the thermal stability and combustion behavior.

## 2 Materials and Methods

### 2.1 Materials

**Figure 1:**
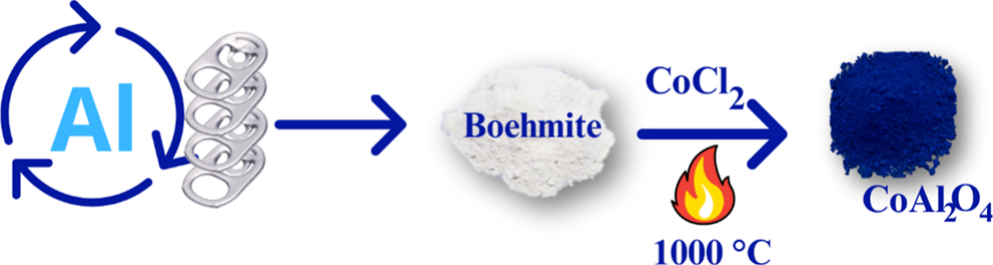
Synthetic process of the flame-retardant pigment.

Aluminum cobalt oxide (CoAl_2_O_4_), used as a synergistic FR additive, was prepared following the synthesis process shown in Figure 1. First, acid digestion (HCl 1.1 mol·L^–1^) was carried out on aluminum can seals, a material used due to the high amount of aluminum in its composition. After the Al^3+^ ions were obtained in the solution, boehmite was precipitated by correcting the pH by adding NaOH solution (2 mol·L^–1^). The flame-retardant pigment is prepared from boehmite following the method described by Horsth et al.^1^ Here, 20% (w/w) of the coloring ion (Co^2+^) was used to synthesize the intense blue pigment. The color is dependent on the coloring ion during the adsorption step by boehmite obtained by recycling aluminum can seals.

The PLA 4032D resin used in this study was supplied by Nature Works LLC (Blair, Nebraska (NE), USA). It presents a high molecular weight (Mn = 119,000 g·mol^–1^ (average molar mass number), Mw 209,000 g·mol^–1^ (average molar mass by weight), and dispersity = 1.9), low d-isomer content (1.4%), a melt flow rate of 7 g.10 min^1^ (measured at 210 °C, 2.16 kg), and a melting temperature (Tm) in the range of 155 to 170 °C. Ammonium polyphosphate (APP 422) was provided by Clariant.

### 2.2 Preparation of PLA Composites

After drying at 70 °C for 24 h to limit PLA degradation during melt processing due to the presence of moisture, PLA, ammonium polyphosphate (APP422), and CoAl_2_O_4_ pigments were melt-blended in a Brabender Plastograph (W50EHT-3, Germany) for 10 min at 200 °C, with rotation rates of 30 rpm for 3 min and 100 rpm for 7 min. The so-obtained blends were thus compressed-molded into plates for Mass Loss Cone (MLC) tests (10 × 10 × 0.3 cm^3^) and specimens for UL-94 tests (12.5 × 13 × 0.3 cm^3^) at 200 °C by using an Agila PE20 hydraulic press. The following three-step pressure program was used for the preparation of the different specimens: first, the sample is deposited for 3 min on the hot part and pressed at 10 bar for 3 min 20 s, followed by three degassing, then it is pressed again at 150 bar for 2 min 30 s, and finally, it is pressed for 5 min in the cold part. The identification label and chemical composition of the studied samples are presented in Table 1.

**Table 1:** Content (Expressed in wt %) of PLA and Additives Used to Prepare the Different Compositions

sample label	PLA (wt %)	APP422 (wt %)	CoAl_2_O_4_ (wt %)
PLA	100		
PLA@APP	80	20	
PLA@APP@CoAl_2_O_4_	80	15	5

Other materials were analyzed to determine the optimal concentrations in this study. Compared with a 10% concentration of APP422, the best performance was observed with the 20% total flame-retardant additives reported in this study.

### 2.3 Methods of Characterization

#### 2.3.1 Thermogravimetric Analyses

The thermal stability analysis of the different additives and compositions was performed using a thermogravimetric analyzer Q50 TA Instrument TGA (New Castle, England) by heating the samples under air from room temperature up to a maximum 800 °C (platinum pans, heating ramp of 20 °C/min, 60 cm 3/min gas flow rate).

#### 2.3.2 Fire Properties

Fire behavior of the different compositions was evaluated by using MLC and UL-94 tests. MLC tests were performed at 35 kW/m^2^ using MLC (from FTT, Ltd., East Grinstead, West Sussex UK), equipped with a thermopile and chimney for heat release assessment, according to the ISO 17554 standard, to determine the peak of heat release rate (pHRR), the total heat release (THR), and the time to ignition. UL-94 vertical burning tests were performed on a FIRE apparatus according to the ASTM D 3801 standard procedure. Samples measuring 125/13/3 mm^3^ were subjected to two flame applications (10 s each). The after flame and afterglow times were measured, and the eventual cotton ignition by flaming drops was recorded.

The CoAl_2_O_4_ sample underwent characterization through powder X-ray diffraction (XRD) analysis using a Bruker D2 Phaser Diffractometer (Berlin, Germany) equipped with a LynxEye high-performance detector and operated at a power of 300 W. Cu Kα emission (λ = 1.5418 Å) was employed during the structure measurements. The oxidation state and composition of the surface chemical elements were assessed through X-ray photoelectron spectroscopy (XPS) utilizing a Versaprobe PHI 5000 instrument (Physical Electronics, Chanhassen, MN, USA) equipped with a monochromatic Al Kα X-ray source. The spectra were analyzed by using CASA-XPS software. The binding energy of the XPS spectra was calibrated using the C 1s peak at 284.6 eV. Raman spectra were recorded using a micro-Raman system (Senterra Bruker Optik GmbH, Massachusetts, USA) with a laser wavelength of 532 nm and a power of 10 mW. Colorimetric analysis was performed on the pigments using a portable colorimeter (3nh, model NR60CP) with a D65 light source (Shenzhen, China).

## 3 Results and Discussion

### 3.1 Characterization of the CoAl_2_O_4_ Pigment

**Figure 2:**
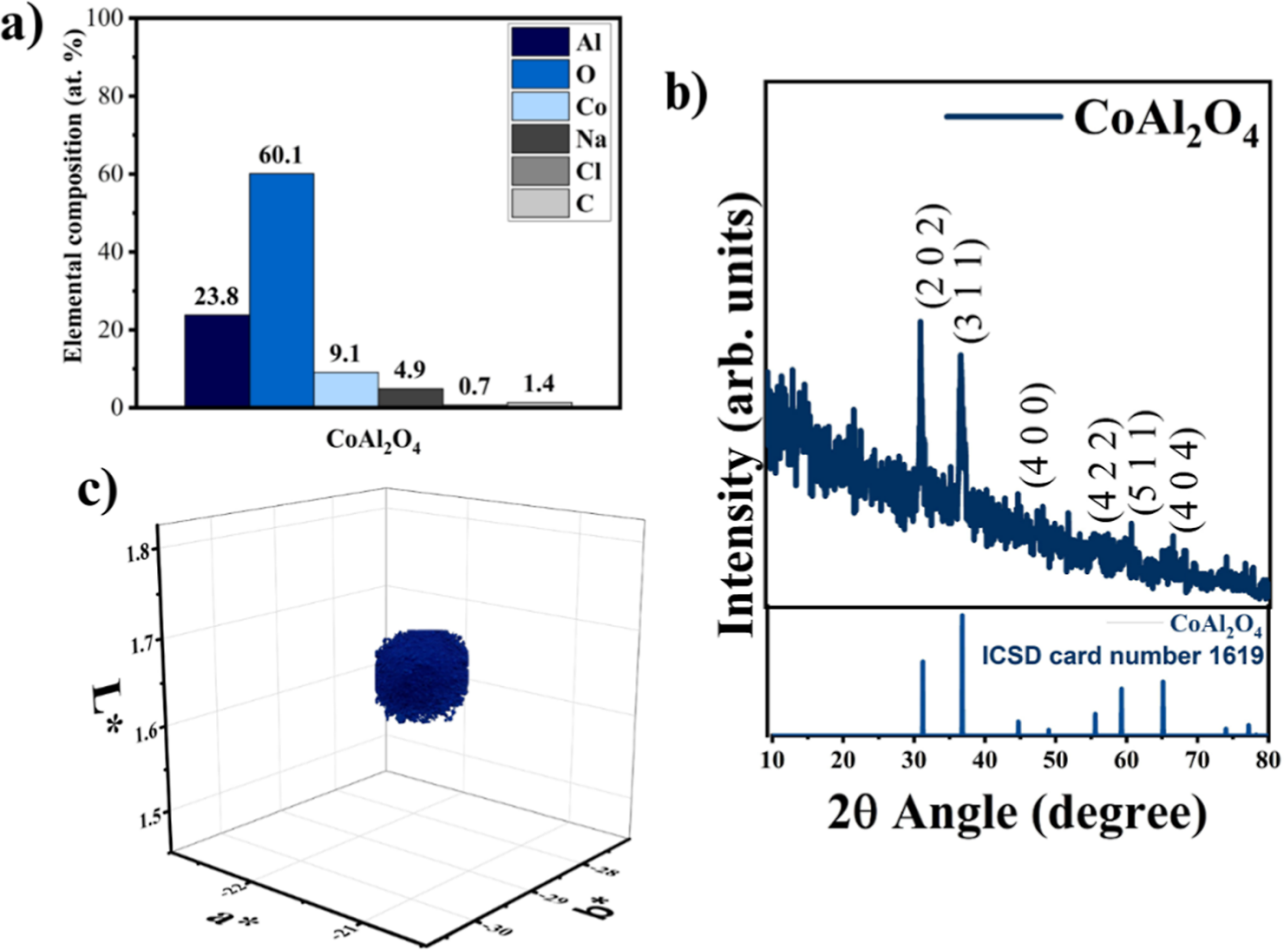
CoAl_2_O_4_: (a) Evaluation of sample composition by XPS, (b) XRD spectrum; (c) Colorimetry CIEL*a*b*.

**Figure 3:**
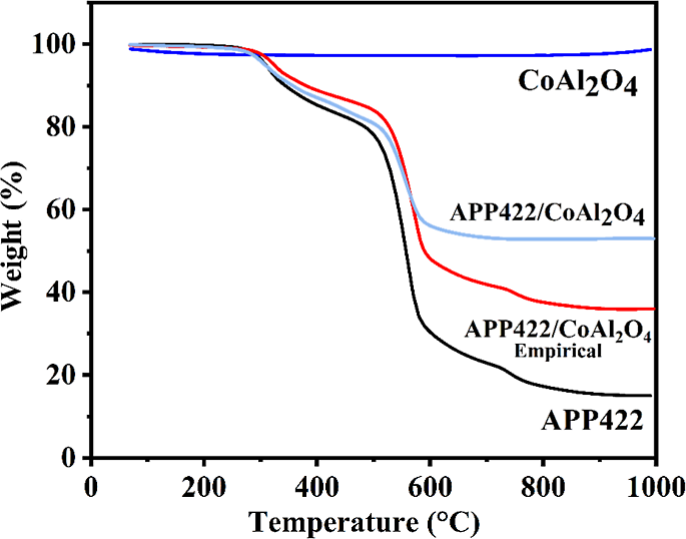
TGA curves of APP422, synthesized CoAl_2_O_4_, their blend (75 wt % APP422/25 wt % CoAl_2_O_4_) at 20 °C/min under air, and empirical curve of the APP422/CoAl_2_O_4_ blend.

XPS analysis was used to evaluate the chemical composition of the synthesized CoAl_2_O_4_ pigment, used as an additive to PLA in combination with APP. As illustrated in Figure 2a, the relative concentration of cobalt is 9.1 atom %, with a ratio of approximately 2.5 in relation to aluminum, in accordance with the anticipated stoichiometry. The presence of Na and Cl originates from the synthesis process. The XRD pattern shown in Figure 2b refers to the crystallographic chart ICSD 1619, characteristic of the diffraction patterns of spinel samples calcined at 1000 °C. The peaks observed at 2θ angles centered at 31°, 37°, 45°, 56°, 59°, 60°, and 65° are assigned to CoAl_2_O_4_. The crystalline structure of the samples belongs to the cubic space group *Fd*3̅*m*.^25^ A pigment is a solid substance characterized by coloration that imparts color to other materials without undergoing significant changes. This color stability is attributed to its robust crystallographic structure, which is characterized by its resistance to temperature changes, chemical reactions, UV light irradiation, or physical stress. This resilience is observed in the spinel structure of CoAl_2_O_4_, found in certain synthetic metal oxides, especially aluminates.^26^ This stability prevents pigments from dissolving upon application, instead resulting in the formation of a colored material. Colorimetry was performed to determine the colorimetric parameters of the CoAl_2_O_4_ pigment (Figure 2c). The analysis indicates that this pigment falls within the blue/green quadrant, exhibiting negative a* and b* parameters. In addition, as a dark pigment, it exhibits low luminosity. The hue value (233.2) indicates pure blue coloration of this pigment. Moreover, the prepared pigment demonstrates highly stable thermal behavior, as evidenced by TGA, which shows no signs of thermal degradation up to 1000 °C (Figure 3, dark blue curve). The subtle weight variation observed for the CoAl_2_O_4_ powder sample is related to the equipment’s baseline, which may result from improper placement of the sample on the instrument’s balance.

**Figure 4:**
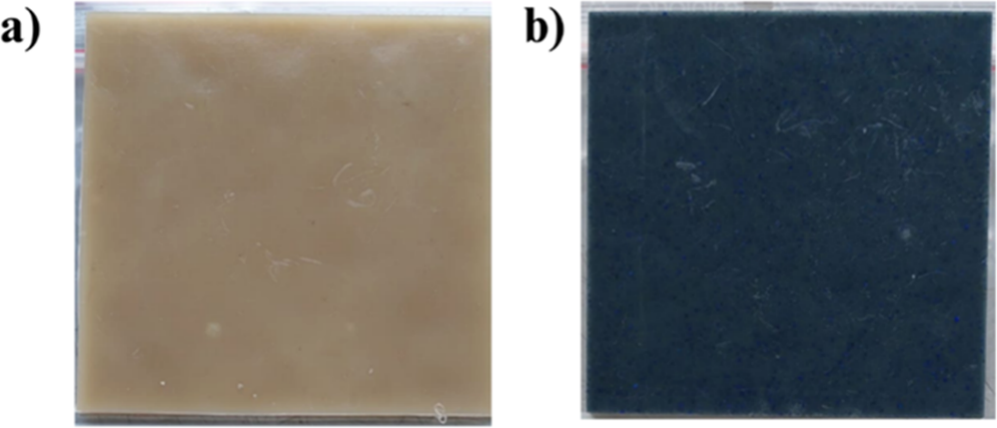
Photography of plates of (a) PLA@APP and (b) PLA@APP@CoAl_2_O_4_.

The CoAl_2_O_4_ pigment was blended with APP422 and incorporated into PLA (see the Materials and Methods). The thermal and fire resistance properties of this composite were evaluated in comparison to PLA and a composite sample containing 20 wt % APP422. From Figure 4, it is noteworthy that the coloring power of the pigment is maintained during the melt processing of PLA. The sample plate displays homogeneous coloration throughout the surface.

### 3.2 Thermal Stability and Fire Properties of PLA/APP422/CoAl_2_O_4_ Composites

**Figure 5:**
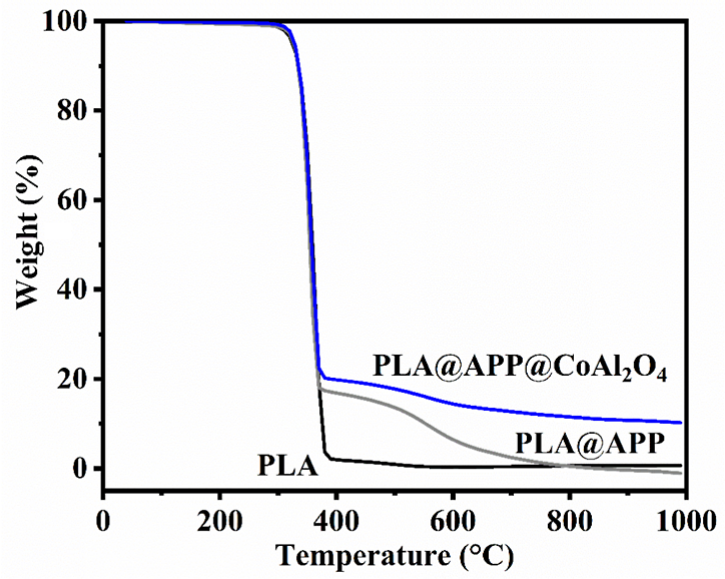
Experimental TGA curves of PLA, PLA@APP, and PLA@CoAl_2_O_4_@ APP.

The effect of incorporating the investigated additives on the PLA thermal stability was assessed by TGA. The curves are shown in Figure 5; pristine PLA undergoes a one-step thermal decomposition, starting around 310 °C and leading to a total decomposition of the material without forming any residue. The incorporation of 20 wt % APP422 (PLA@APP) does not induce any premature thermal degradation of the polyester matrix, and the composite (PLA@APP) thermal degradation starts at the same temperature as for neat PLA, which is 400 °C. However, for the PLA@APP composite, the degradation occurs in two steps. The first one leads to the formation of a char that totally decomposes during the second degradation step. The combination of both additives leads to a notable behavior: (1) no premature thermal decomposition induced by CoAl_2_O_4_ and (2) a more intensive formation of char residue, demonstrating higher thermal stability until 1000 °C under oxidant conditions. This behavior can be attributed to the synergy between the swelling agent APP422 and the thermally stable pigment CoAl_2_O_4_ at this temperature.^27^ To determine whether the enhancement of the char amount and its thermal stability has additively or synergistically occurred as a consequence of the presence of both CoAl_2_O_4_ and APP422, we calculated the additive TGA response (empirical curve) and compared it with the experimental curve of the APP422/CoAl_2_O_4_ powder blend at the same ratio as in the composite. The empirical curve was calculated by considering the TG curves of APP422 and CoAl_2_O_4_ under air at the same relative content as in the composite (75 wt %/25 wt %). Figure 3 presents experimental and empirical curves of the APP422/CoAl_2_O_4_ blend (75 wt %/25 wt %). These curves highlight a synergistic effect, since the final residue generated during the thermal decomposition of the powder blend is higher than the empirical one. This synergistic effect may be due to the reaction between the CoAl_2_O_4_ and APP422 additives induced by high temperature. This indicates an interaction between the two additives, leading to the formation of a thermally stable residue. Such a synergistic effect could explain the enhancement of the thermal stability of the PLA composite filled with both additives. Again, the difference between the empirical and experimental char amounts is significant. Thermal degradation of the APP422 and CoAl_2_O_4_ mixture leads to the formation of a final residue of around 53%, whereas the final residue expected when the additives degrade separately (theoretical curves) should be around 35%. Accordingly, the presence of CoAl_2_O_4_ has a positive effect on APP422, whose thermal degradation leads to more residue and better thermal stability in the presence of CoAl_2_O_4_.

### 3.3 Fire Behavior

**Figure 6:**
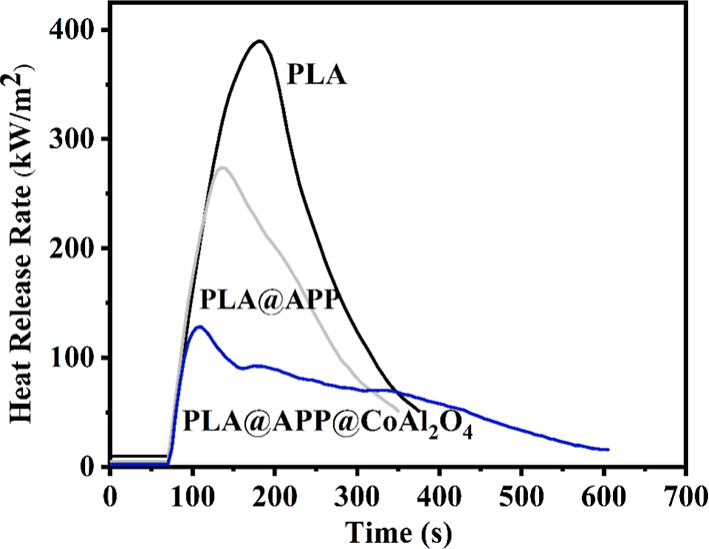
HRR curves of PLA and its composites.

The positive effect of CoAl_2_O_4_ on the amount of char produced and its thermal stability during the thermal degradation of PLA containing APP422 can also be appreciated during the MLC test. Figure 6 presents the heat release rate HRR curves versus time for neat PLA, PLA containing 20 wt % APP422, and the composite containing the combination of 15 wt % APP422 and 5 wt % CoAl_2_O_4_. The key parameters of fire retardancy of PLA and PLA composites are listed in Table 2. The combustion of PLA starts at 68 s, consuming all of the material with a pHRR of 380 kW/m^2^, reached after 200 s.

**Table 2:** Content (Expressed in wt %) of PLA and Additives Used to Prepare the Different Compositions

sample label	TTI (s)	pHRR (kW/m^2^)	pHRR reduction (%)	THRR (MJ/m^2^)	THRR reduction (%)
PLA	68	380		61.2	
PLA@APP	70	270	–30	41.4	32.35
PLA@APP@CoAl_2_O_4_	70	120	68.4	29.2	52.3

The presence of APP422 alone improves the flame-retardant behavior of PLA due to the formation of a char residue during combustion. This residue acts as a thermal shield and a physical barrier, limiting the diffusion of heat through the material. Thus, the heating of unexposed layers and the volatilization of combustible fragments reduce the fuel supply of the gaseous phase. This results (Figure 6) in a significant reduction in the pHRR, from 380 to 270 kW/m^2^, and without any reduction in the resistance to ignition since the ignition time remains stable at 70 s. When APP422 is combined with CoAl_2_O_4_ at a constant overall incorporation rate (20 wt %), the efficiency of the flame-retardant system increases dramatically, and the pHRR drops sharply at 120 kW/m^2^, corresponding to a pHRR reduction of about −68.4%, whereas it is only −30% when APP alone is used. A similar trend is also observed for THR, which is significantly reduced when the blend contains both additives (−52.3%), whereas with APP alone, the reduction is only −32.35%.

**Figure 7:**
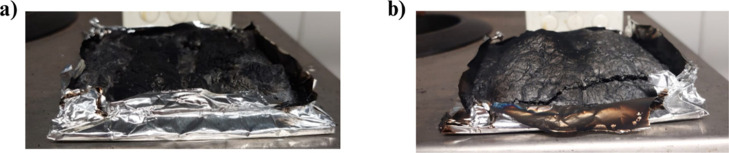
Pictures of the char formed during MLC of (a) PLA@APP and (b) PLA@APP@CoAl_2_O_4_.

This superior effect is explained by the formation of a charred layer that rapidly covers the entire burning plate. This conclusion is based on visual observations from the MLC test. We observed that as soon as the heating element was exposed, small charred islands formed and connected rapidly, covering the entire surface of the plate. The formation of this type of residue enables the material to be more effectively insulated in the early stages of combustion. Moreover, we observe that the rate of heat release remains the same for all three compositions but that the pHRR is quickly reduced as the residue covers the entire plate more quickly when APP422 is combined with CoAl_2_O_4_. Moreover, we can observe that the char obtained when both additives are incorporated into PLA presents a cohesive expanded structure (Figure 7).

The three composites were also tested using the UL-94 test, an internationally used flammability test standard for plastic materials developed by Underwriters Laboratories (UL), a global safety certification organization. The primary purpose of the UL-94 test is to evaluate the flammability of plastic materials when exposed to an open flame.^28^

It is important to note that in the UL-94 tests, no improvement in rating was obtained despite the development of this synergistic effect between the two additives. Unfilled PLA obtains no classification at this test, while PLA containing 20 wt % APP422 obtained a V2 rating, which was maintained in the case of the composite containing the two additives despite the decrease in APP422 content, suggesting that to improve the test score, higher concentrations of additives should be tested.

### 3.4 Structural Characterization of the Combustion Residue

**Figure 8:**
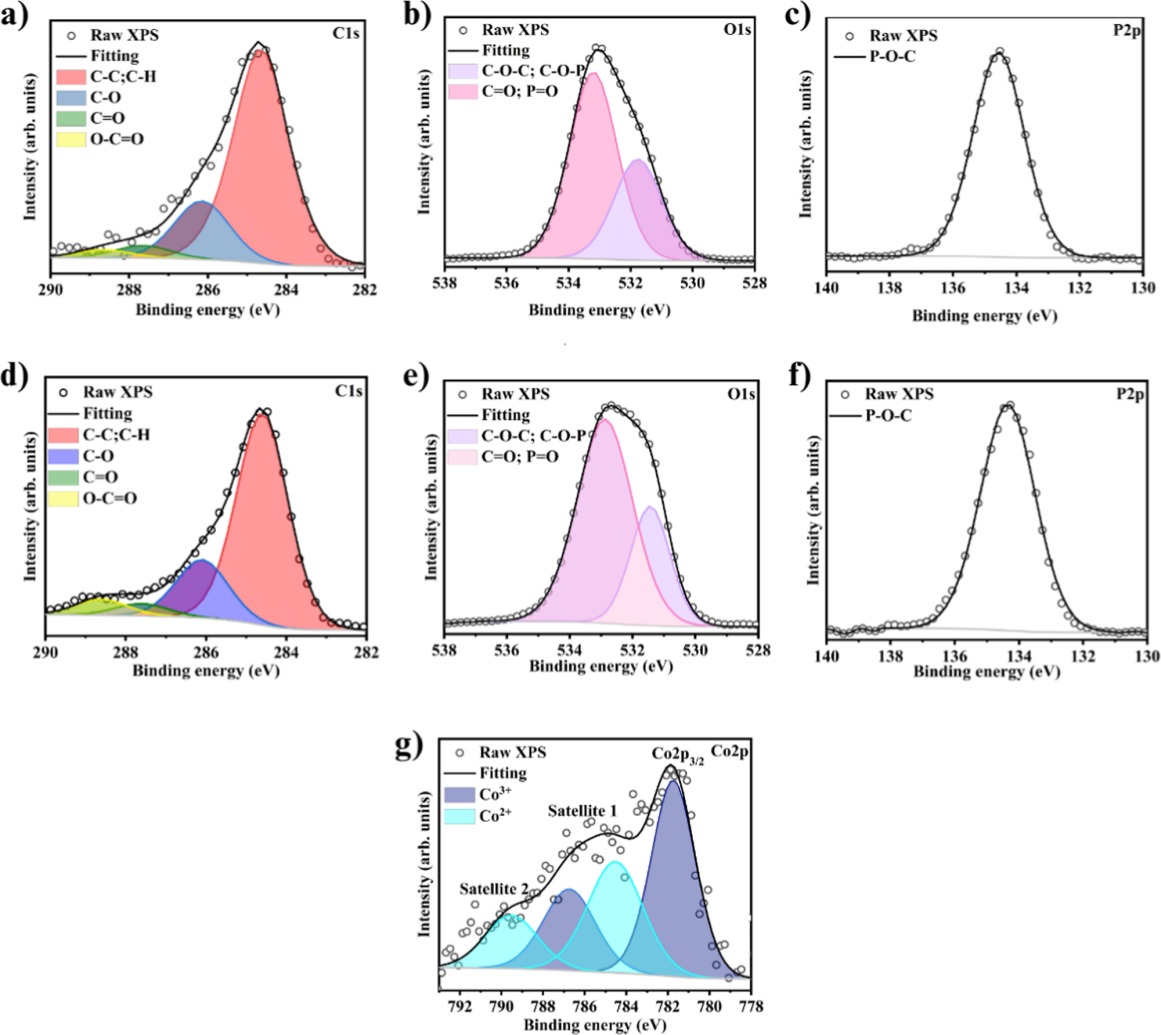
XPS spectra of residue char. (a–c) PLA@APP, (d–g) PLA@APP@CoAl_2_O_4_.

The chemical structure of the combustion residue was analyzed by using XPS and Raman spectroscopy. XPS was used to evaluate the elemental composition of the char residue (Figure 7). The curving fitting of the C 1s peak shows two main components centered at 284.7 and 286.2 eV, corresponding to C–C, C=C, and C–O, respectively.^29^ In Figure 8d, the C 1s spectrum of APP@CoAl_2_O_4_@PLA reveals an additional component at 288.7 eV, indicative of the presence of C=O bonds.^30^ The O 1s spectrum in Figure 8b,e exhibits two components, indicating the presence of oxygen in different chemical environments within the material, components at 533.0 and 531.5 eV, attributed to C–O–P, C–O–C groups, and O= in C=O and P=O groups, respectively.^31^ The P 2p spectra in Figure 8c,f are assigned to P–O–C,^32^ suggesting that phosphorus (P) is mainly present in the structure of P–O–C within the carbon residue. The Co 2p_3/2_ spectrum (Figure 8g) was reproduced by two components,^33,34^ referring to the presence of Co^3+^ and Co^2+^.^35,36^ The satellite shifted by 6 eV from the primary component, and the high-energy component is further evidence of the presence of Co^2+^ species due to the reduction of Co^3+^ to Co^2+^ present in octahedral sites.^36−39^ The reduction of Co^3+^ to Co^2+^ shows that cobalt ions react with P through an oxidation/reduction process. Similar behavior has been reported in the case of Fe_2_O_3_ and red phosphorus combination.^40^ In this case, the authors evidenced the reduction of Fe_2_O_3_ into Fe_3_O_4_, which was attributed to the oxidation of P by iron, leading to superior flame-retardant action.

**Figure 9:**
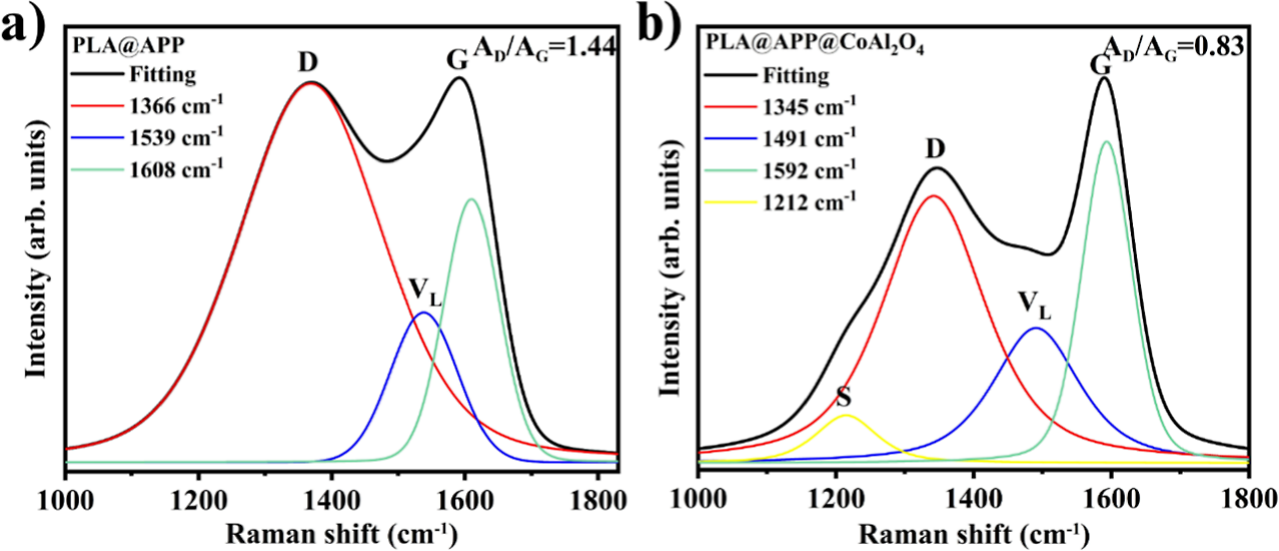
Raman spectra of residue char for (a) PLA@APP and (b) PLA@APP@CoAl_2_O_4_.

**Figure 10:**
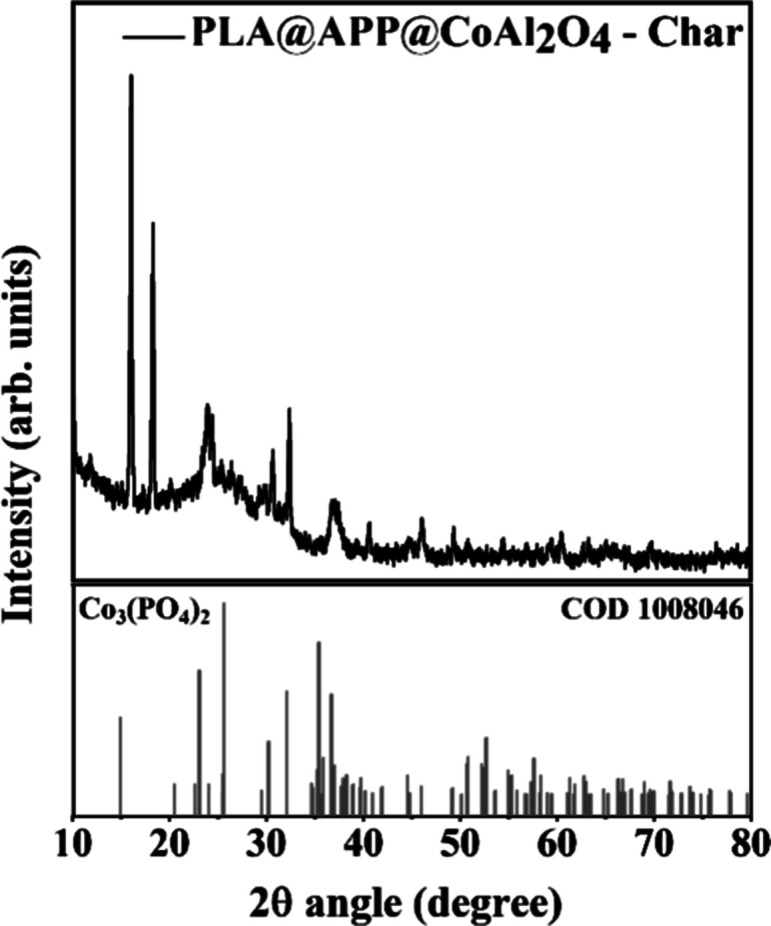
Char diffractogram with the Co_3_(PO_4_)_2_ phase indexed by the crystallographic chart ICSD 4268.

The structure of the char layer was additionally characterized through Raman spectroscopy analysis and XRD. In Figure 9a,b, the Raman peaks centered at 1345 cm^–1^ for the D band and 1590 cm^–1^ for the G band are attributed to amorphous and graphitized carbon, respectively.^41^ The band centered around 1345 cm^–1^ is attributed to the D band, resulting from the vibration of carbon atoms with dangling bonds at the planar ends of disordered graphite or glassy carbons.^42,43^ The band centered at around 1590 cm^–1^ corresponds to the G band, originated by the vibration of sp^2^-hybridized carbon atoms in the graphite layer. Furthermore, the G band is associated with the E_2g_ mode of hexagonal graphite.^42,43^ Both spectra show the presence of the V_L_ band, often related to aromatic carbon vibrations.^44,45^ PLA ((C_3_H_4_O_2_) simplified representation of a lactic acid unit) and APP (ammonium polyphosphate) when subjected to high temperatures can undergo degradation reactions that result in the formation of pyrolysis products, including aromatic compounds and other degradation products.^46^ The low-frequency component of the D, S-band centered at 1212.9 cm^–1^ can be identified as a peak in the phonon density of states. Its appearance can be attributed to the incorporation of cobalt, which leads to changes in molecular vibrations and the structure of the carbonaceous matrix.^47^ The displacement of the G-band induced by cobalt insertion indicates fracture of agglomerates and disordered chains, which is accompanied by graphitization of the char.^47^ The A_D_/A_G_ ratios of char residue decreased from 1.44 (PLA@APP) to 0.83 (PLA@APP@CoAl_2_O_4_), indicating an increased graphitization degree of the char in the presence of the CoAl_2_O_4_ pigment. This again confirms the positive impact of the cobalt aluminate in forming a high-quality char layer, as shown in Figure 7, together with the photographic record of the prepared composite, in agreement with data reported in the literature.^48^ The diffractogram (Figure 10) obtained for the sample char demonstrates the dominance of the Co_3_(PO_4_)_2_ phase indexed by crystallographic chart ICSD 4268. Transition metal phosphates show remarkable structural stability over nonoxides, especially in oxidative environments.^49^ The flexible coordination features of phosphate groups could stabilize the intermediate states of the transition metal centers.^49^ This justifies the high thermal stability observed in the char formed.

**Figure 11:**
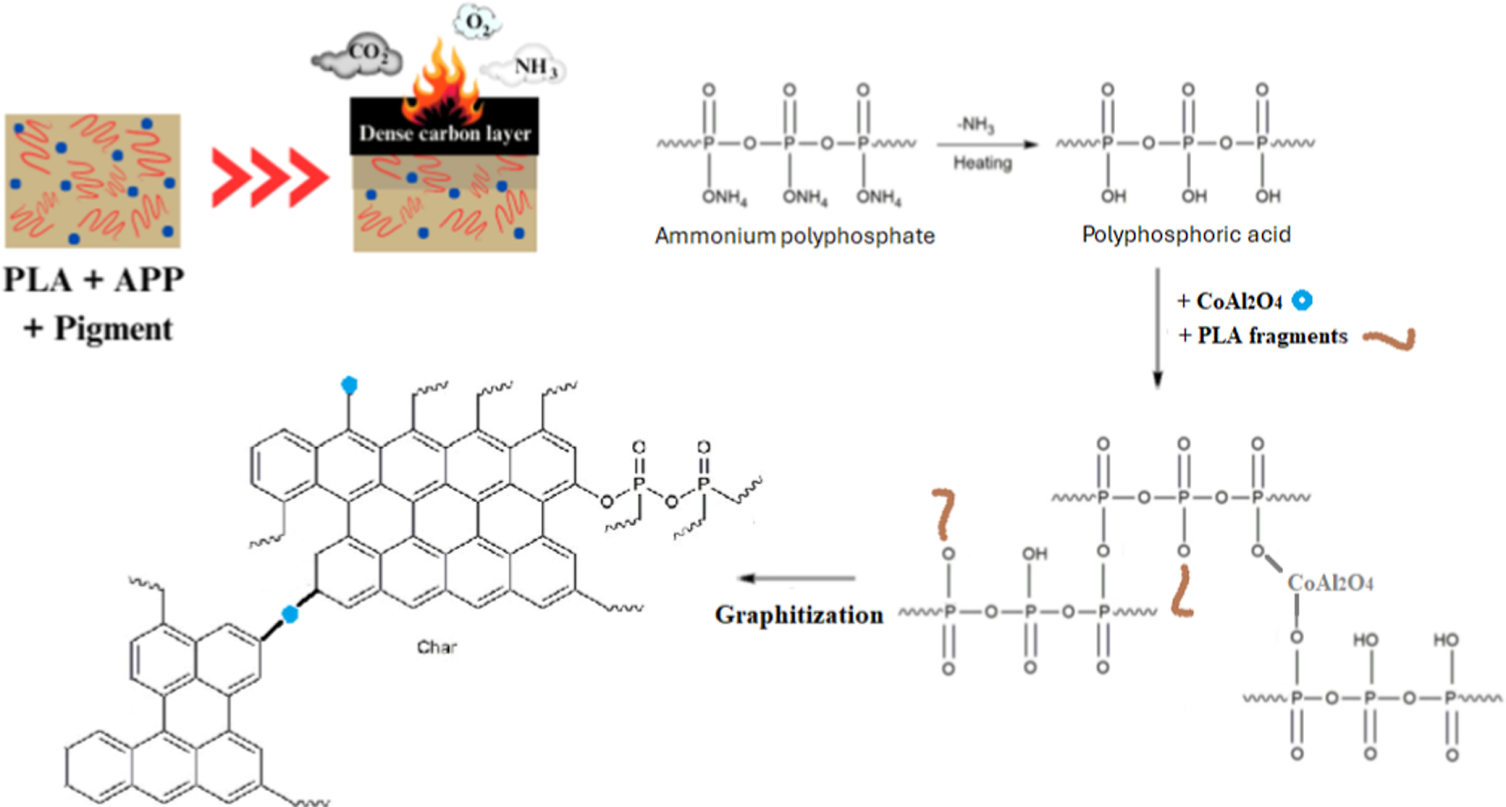
Proposed flame-retardant mechanism for PLA@APP@CoAl_2_O_4_ composition.

Figure 11 proposes a mechanistic representation of possible events taking place in the system to explain the superior FR effect of PLA@APP@CoAl_2_O_4_. The presence of CoAl_2_O_4_ increases the efficiency of APP by improving the amount and the quality of the char formed during combustion, since CoAl_2_O_4_, when used independently, lacks the capacity for the formation of a protective char layer. TGAs highlighted the increase in char amount when these two elements are combined, while Raman analyses revealed a higher degree of graphitization in the char formed during combustion of the PLA/APP/CoAl_2_O_4_ composition. Improving char quality and its thermal resistance is key to enhancing PLA fire behavior. This is due to the significant reactivity between PLA and CoAl_2_O_4_, which was evidenced by XPS analyses notably through the reduction of Co^3+^ to Co^2+^ that evidenced that cobalt ions react with P through an oxidation/reduction process and also by the formation of the Co_3_(PO_4_)_2_ crystalline phase evidenced by XRD analyses. The combination of these two elements thus enables the rapid formation of a three-dimensional network, thanks to the condensation reactions of poly(phosphoric acid), which are accelerated and stabilized by its interaction with CoAl_2_O_3_. This results in a char presenting a high degree of graphitization and therefore better thermal resistance.^50^

## 4 Conclusions

In conclusion, this preliminary study demonstrates the interest of CoAl_2_O_4_ obtained by recycling metallic aluminum waste as a pigment and a synergetic flame-retardant additive. First, the blue pigment, prepared from the recycled material, is thermally stable up to 1000 °C, and its incorporation into PLA does not lead to any polymer degradation during thermal analysis. When combined with APP422, CoAl_2_O_4_ induces significant enhancement in both quantity and thermal stability of the decomposition residue, avoiding thermal degradation when exposed to fire, indicating the possibility of application as a material for 3D printing. Structural analyses carried out on the char obtained during the combustion test revealed an oxidation–reduction interaction between Co^3+^ ions and APP422, which induces the formation of a Co_3_(PO_4_)_2_ phase, likely responsible for improving the thermal stability of the produced char. Raman analysis also reveals a significant enhancement in the graphitization degree of the char in the presence of CoAl_2_O_4_.

This study not only provides a foundation for the utilization of recycled aluminum waste in high-performance applications but also underscores the significance of sustainable material development within the framework of flame-retardant technologies. By integrating the recycled material into functional composites, this approach aligns with the principles of a circular economy while offering practical solutions for fire safety in modern polymer-based applications, particularly in the rapidly evolving field of 3D printing.
